# Advancing environmental risk assessment for transgenic biofeedstock crops

**DOI:** 10.1186/1754-6834-2-27

**Published:** 2009-11-02

**Authors:** Jeffrey D Wolt

**Affiliations:** 1 Department of Agronomy and Biosafety Institute for Genetically Modified Agricultural Products, Iowa State University, Ames, IA 50011, USA

## Abstract

Transgenic modification of plants is a key enabling technology for developing sustainable biofeedstocks for biofuels production. Regulatory decisions and the wider acceptance and development of transgenic biofeedstock crops are considered from the context of science-based risk assessment. The risk assessment paradigm for transgenic biofeedstock crops is fundamentally no different from that of current generation transgenic crops, except that the focus of the assessment must consider the unique attributes of a given biofeedstock crop and its environmental release. For currently envisioned biofeedstock crops, particular emphasis in risk assessment will be given to characterization of altered metabolic profiles and their implications relative to non-target environmental effects and food safety; weediness and invasiveness when plants are modified for abiotic stress tolerance or are domesticated; and aggregate risk when plants are platforms for multi-product production. Robust risk assessments for transgenic biofeedstock crops are case-specific, initiated through problem formulation, and use tiered approaches for risk characterization.

## Review

Agricultural biofeedstocks are critical for decreasing petroleum dependence through sustainable biofuels production. Continued rapid improvements in both biofuel resources and processes are needed if agricultural biofeedstock crops are to significantly address concerns about the depletion of fossil fuel reserves, domestic energy security and greenhouse gas emissions as contributors to climate change [1]. Various perspectives are advanced as to whether to develop dedicated bioenergy crops from non-food crops [2] or to use food crops as multi-platforms for food, feed and fuel production [3], as well as the appropriate technologies, including transgenics, for biofeedstock development [4]. The current first generation biofeedstock crops represent modification and the use of food-based grains for biofuel production. These will be largely supplanted by second generation crops representing specialized industrial oilseed crops and the utilization of lignocellulosic crops and crop residues [5]. Unless, and until, third generation technologies using algae and bacteria become a reality, plant-based agriculture - with its attendant tradeoffs regarding land use alternatives and the balance of needs for food, feed and fuel production - will remain the leading opportunity for biofuel production.

Implicit in the successful movement towards a biofuel future is the use of modern biotechnology, including transgenics, as an enabling technology [1,5,6]. Transgenics - the use of genetic engineering to introduce genetic information across species - will be critical to near-term adaptation of food crops for biofuels production by, for instance, improving yields and processability for starches and oils, affecting value-added changes in crop composition, or improving stress tolerance to assure more predictable biomass streams for processing. In addition, over the longer term, biofeedstock crops will require the domestication of semi-domestic or wild plant species to accommodate wide-scale agronomic production. Transgenics represents a leading method for the timely development and deployment of these unique biofeedstock crops [5].

As for any technological innovation, the use of a particular transgenic concept for biofeedstocks production must undergo commercial, regulatory and public scrutiny in terms of its impact on the environment, human and animal health and sustainability [6]. Such considerations are approached from within both a narrow regulatory context (that is, the view of how a particular transgenic is scrutinized within existing regulatory statutes) and a broader decision context (as, for instance, in determining which technologies will be targeted for development by private enterprise and the public sector). An array of decision-making tools, such as risk assessment, life cycle analysis and sustainability evaluation, are available for focused assessment and broader decision-making [7]; but of these, risk assessment lies along the critical path for the acceptance and development of transgenic biofeedstock crops. This is because regulatory guidance exists, or is rapidly emerging, that identifies risk assessment as the method of choice for considering risks associated with transgenic plants and the means by which risks may be managed [8-10]. Frameworks for risk assessment have been developed and implemented at both the national and international level [11-14] with their application most strongly focused on chemical stressors (see, for instance, [15]). From the perspective of these frameworks, the US National Research Council has considered the improvement and utility of risk assessment paradigms [7]. Among findings are the need to better define the risk assessment process through emphasis on the initial problem formulation and scoping; increased emphasis on design of the risk assessment; more clearly addressing uncertainty and variability; appropriate selection and use of assessment defaults; and improving the interface between risk assessment and risk management in terms of risk options and mitigation measures [7].

Despite their more recent development, risk assessment frameworks for transgenic plants are being widely implemented [8,16-20] and numerous representative cases provide evidence of their utility as well as the current limitations [21-28]. Importantly, many of the aforementioned broader needs for improved risk assessment are recognized for the more specific case of transgenic plant risk assessments as well; as for instance, the need to properly plan the risk assessment through appropriate problem formulation [29,30]. Advancing risk assessment for transgenic plants used as biofeedstock crops represents a critical element to rapid and cost effective development of plant biofuel resources. Here we discuss the needs for advancing transgenic plant risk assessment by appropriately interfacing science with the needs for decision-making. Aspects of risk assessment are also illustrated relative to specific types of biofeedstock crops.

## Integrating science and decision-making through risk assessment

### The risk assessment process

**Figure 1:**
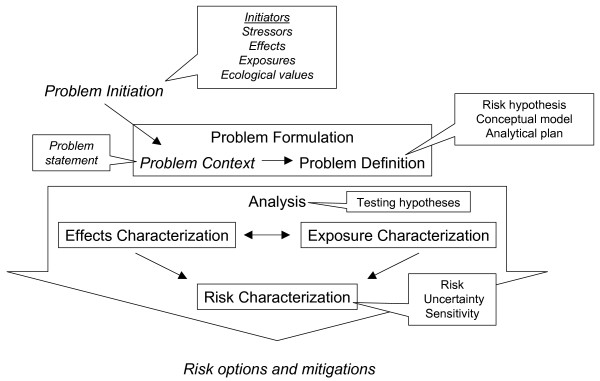
**The interface of science and decision-making: risk assessment within a risk analysis framework**. Italicized components are largely risk management actions.

In consideration of the fact that risk is the joint probability of exposure and the consequence of exposure, the conventional risk assessment process describes exposure and its consequence (an adverse effect or harm) in four steps: hazard identification, dose-response, exposure characterization and risk characterization [11]. For transgenic plants, these steps are more aptly termed problem formulation, effects characterization, exposure characterization and risk characterization. Risk is assessed through a science-based process that integrates with risk management to facilitate informed decision-making (Figure 1). Although risk assessment is often illustrated as a linear process (see, for instance, [13]), the process in practice is non-linear, recursive and iterative such that the sequence in which aspects of the assessment are addressed varies depending the knowledge needed to analyse a given case.

Evidence of hazard - the recognized potential of a substance to cause harm to human health or the environment - is not common for transgenic plants and, therefore, the broader consideration of potential harms warranting evaluation is identified through problem formulation (see following discussion). This approach is in keeping with the recognition that problem formulation and scoping (inclusive of hazard identification) needs increased emphasis in risk assessment [7]. Similarly, because hazard is generally absent, consideration of a threshold effect through dose-response is insufficient for the needs of transgenic plant risk assessments. An exception is for cases of non-target effects to sensitive insects from plants expressing insecticidal toxins from *Bacillus thuringiensis *[31,32]. Thus, for transgenic biofeedstock crops a more holistic effects characterization considers the harm arising from direct effects (dose-response) as well as broader ecological metrics (an example being indirect effects on herbivory for the case of *Bt *crops [31]).

The transgenic plant risk assessment adheres to a weight-of-evidence approach that considers comparative risks. The weight-of-evidence approach allows for the consideration of how, for a particular case, the preponderance of evidence identifies risk to human health or the environment. This directs decision-making away from a narrowly focused evaluation of a specific endpoint and, instead, considers risk within a fuller ecological context. A sequential lines-of-evidence approach [33] provides a robust means to address risk, which for transgenic plants is represented in tiered analytical schemes [20].

Implicit to transgenic plant risk assessment is a case-specific consideration developed on the basis of comparability [8]. The principle of comparability considers the attributes of a specific transgenic plant relative to a baseline understanding of the degree that the plant and its alteration are familiar. Further, it considers the equivalence of attributes of the transgenic plant relative to comparable non-transformed plants (comparators) under conditions that represent the intended receiving environment. Establishing familiarity identifies the appropriate baseline for the risk assessment so as to focus on the biologically relevant change which is manifested in the transgenic plant. Determination of the appropriate comparator is very much dependent on the problem that is formulated and may include considerations such as typical crops and cropping conditions in the use environment or genetic background of the modified crop.

For biofeedstock crops equivalence for many attributes of the plant is not intended. For instance, desirable changes may represent altered profiles of oils or enzymes, altered metabolic profiles for stress tolerance, or widely altered phenotypes of wild or semi-domestic species so as to better respond to agronomic management. For any of these concepts the consideration of equivalence still holds with a focus on the intended changes and establishing for all other plant attributes their equivalence to the non-modified comparator.

The phenotype is generally recognized as the key to understanding comparability on the basis of familiarity and equivalence relative to a baseline. Phenotype refers to observable characteristics of an organism described as physical or biochemical traits. What constitutes a phenotype should reflect a biologically relevant level of detail consistent with the particular risk comparison being made. Through a consideration of a specific transgenic plant (an event) with respect to appropriate comparators, comparability describes the intended change brought about by the transformation and establishes that the transformation is limited to the intended phenotypic change. Thus, the transgenic plant phenotype is compared to baseline phenotypes with a focus on the difference found through the comparison. When the degree of equivalence is established, the subsequent risk assessment can focus on the change. The nature of phenotyping and the way that change is represented through genetic engineering of plants will be an area for continued clarification as scientists anticipate novel innovations such as transcription factors, changes in metabolic profiles, or RNA interference.

A further aspect of the comparative risk assessment for biofeedstock crops is the consideration of alterations in agroecosystems and their management when current generation food crops are supplanted by biofuel crops. This will initially represent gradual changes, for instance with respect to maize (*Zea mays*) stover recovery and use for energy production or through crop use for multi-product production (food/feed, biofeedstock, and value-added by-products). However, it will also entail substantial shifts in production systems, as when annual row crop production shifts to perennial biofeedstock production. In these instances, the risk assessment process can be applied within a decision-making context to better understand the risks (and/or benefits) arising from replacement of conventional crops and cropping systems with new technologies, including plants transformed through transgenics. The principle of comparability will consider biofeedstock crops and cropping systems relative to the baseline of existing crops and their management as the comparator.

### Problem formulation: context and definition

Problem formulation is the initial step in risk assessment in which risk assessors work in collaboration with risk managers to arrive at an actionable plan for conduct of a case-specific risk assessment. The problem formulation phase has long been a part of the ecological risk assessment process [13] and problem formulation and scoping is now more widely recognized as an integral step to advancing sound practices in risk assessment [7]. The problem formulation represents a non-linear process of interaction and iteration [12].

Problem formulation has been widely considered from the perspective of transgenic plant risk assessments [20,29,30,34] as a means to identify the nature of questions that are germane to a given case of risk assessment and to arrive at a practicable analytical plan for risk characterization. It is the first step in risk assessment where policy goals, scope, assessment endpoints and methodology are developed into an explicitly stated problem and an approach for analysis [30]. For the transgenic plant risk assessment, problem context and definition together comprise the problem formulation and scoping element of the risk assessment.

Problem context represents the activity that establishes the parameters for the risk assessment [30]. The consideration of problem context is driven largely by the recognized needs for the risk assessment as elaborated by the risk manager. This planning and scoping exercise should distill to a concise summary statement that defines the specific concerns for the risk assessment and the general scope of the subsequent analysis [7]. For instance, the following problem statement was used by Wolt *et al*. [23] in formulation of an analytical approach for investigation of *Bt *maize pollen effects on monarch butterfly, 'The utility of corn expressing Cry1A(b) protein arises from the toxicity of the expressed protein to a specific lepidopteron pest, European corn borer, which is of economic importance in corn production. Because this plant-expressed *Bt *protein is active against lepidopteron species, an assessment of the risk to non-target lepidopteran species inhabiting corn production systems is warranted.' The problem statement focuses a broad-based concern (impact of *Bt *maize pollen) to a specific consideration (maize expressing Cry1A(b) protein and non-target lepidopteron species inhabiting maize production systems) and, thus, distills the problem to an analytically tractable form.

Problem definition proceeds from the problem statement to the identification of postulated significant risks that warrant further analysis for a particular case and leads to a specific analysis plan [30]. The problem definition is that facet of problem formulation which develops the specific technical details for the assessment that is to be conducted and, therefore, represents the design stage for the risk assessment [7].

### Designing the risk assessment

The problem formulation will, ideally, develop a concise problem statement, a risk hypothesis, a conceptual model and an analysis plan. Together, these elements represent the design for the risk assessment and set the bounds for the risk characterization and the way that risk findings will be reported and considered.

The problem statement is an outcome of the consideration of problem context and translates a broad regulatory need to a specific statement of need/intent for the risk assessment which is further clarified and articulated as a risk hypothesis. A risk hypothesis represents an assumption regarding the cause-effect relationships among attributes of the risk characterization, including sources, exposure routes, endpoints, responses, and measures relevant to the risk assessment [13]. The risk hypothesis developed in the problem formulation phase is represented as one or more experimental hypotheses which are tested in the analytical phase of the risk assessment. The way the risk hypothesis is stated influences how the risk assessment will be conducted and the findings interpreted. The risk hypothesis may be initiated from consideration of stressors, effects, exposure, or ecological value (Appendix 1).

The conceptual model and analytical plan are critical products coming from the problem formulation [7]. The conceptual model describes key relationships between a transgenic plant occurring in the environment and its linkages to an assessment endpoint that is characteristic of the adverse human health or environmental consideration which has been identified as the focus of the risk assessment. Along with the risk hypothesis, it sets the problem in perspective and establishes the proposed relationships that need evaluation. Vagueness in the development of the conceptual model leads to uncertainty in subsequent risk findings. The conceptual model should explicitly recognize the assessment endpoints that have been established, the entities of value (individuals, populations, or systems to be protected) and their measurable attributes. It can control for both variability and uncertainty in the risk assessment by defining boundaries for the assessment.

The analysis plan is a plan of work that outlines the analytic and interpretative approaches which will be used to characterize risk. Measures of effect (assessment endpoints) should be established that represent measurable changes relevant to the risk characterization. For instance, if the effect measure relates to a toxicological hazard, an acute effect concentration (EC_50_) or a chronic no observed adverse effects concentration (NOAEC) may be specified as the relevant effect measurement. Secondly, measures of exposure in the environment are described through a causal pathway to contact or co-occurrence of the transgenic plant with the entity of value. These measures of stressor exposure are most often expressed as estimated environmental concentrations (EEC) that are developed on the basis of the casual pathway for exposure. Oftentimes, the analysis plan will also be concerned with measures of system or receptor characteristics. These are characteristics of the ecosystem that influence the behaviour, location, life history characteristics or system attributes relevant to the entity of value. For instance, in the case of non-target risks, this may involve the determination of the abundance and distribution of the entity of value (for instance, a beneficial organism) at a relevant life stage within a landscape or region.

Finally, the analysis plan will establish the appropriate risk formulation to be considered in the risk characterization. The risk formulation represents the way in which the exposure measurement is related to the effect measurement. In its simplest form the risk formulation may be a ratio (a risk quotient, RQ) of exposure to effect measurements (for instance, RQ = EEC/EC_50_). Stochastic risk assessments more frequently compare exposure exceedance probabilities to an effect measurement; as for instance the percent of occasions where exposure exceeds the NOAEC for the entity of value. In many instances, there may be an established regulatory threshold for concern that defines both the appropriate risk formulation as well as the result that would trigger regulatory concern. For instance, in the case of a first tier assessment for transgenic plant effects on non-target arthropods, an RQ < 0.1 would be considered evidence of no likely harm [35].

### Iterative nature of risk assessment: defaults and tiers

The concept of an iterative or tiered system of both testing and assessment is a critical aspect of environmental risk assessment and is used in certain aspects of food safety assessment as well. A tiered framework provides a common approach for selection of relevant studies; it organizes and guides the studies and associated assessments that are required during the regulatory process; it potentially eliminates unneeded studies; and it allows comparative assessments among different technologies [35]. A well-described tiered process establishes default actions and conditions for the design and conduct of a risk assessment [7] and, thus, represents a key tool for the development of a case-specific analytical plan. A tiered testing framework is intended to be flexible to address changing assessment needs on a case-by-case basis. This is because of the ability to acquire new data and update, or iterate, the problem formulation and subsequent risk assessment, providing needed flexibility to address new or changing aspects of risk [30]. There are well-developed tiered testing schemes for conventional pesticides [36,37] and tiered approaches have been described in terms of their relevance for some aspects of transgenic plant food safety and non-target organism risk assessments [20,26,35,38] and more generally [8,39,40]). A limitation of these tiered schemes for transgenic plants is the near-exclusive focus on stressor-initiated or effects-initiated problems, with limited consideration of risk assessments which may be initiated from the standpoint of exposure or ecological value (Appendix 1).

The analytical phase of any risk assessment is initiated with lower tiered testing which conservatively addresses broad questions using simple experimental designs. Any subsequent tests at higher tiers are more realistic and complex. Higher tiers of testing are triggered within the risk assessment process when there is recognition that the degree of potential harm or analytical uncertainties requires more exacting determination of probable risk. Since higher tiered tests are only prompted by the risk assessment process, an iterative approach effectively focuses consideration to the most relevant concerns and conserves time resources. Furthermore, to effectively address risk assessments which may initiate from a variety of concerns, the tiered scheme must allow for a non-linear approach, so as to allow the most relevant lines of evidence to be brought forward. For instance, Rosi-Marshall *et al*. [41] developed a logical conceptual model for understanding the risk for addressing an exposure-initiated consideration (plant residues of transgenic crops occurring in waterways) but followed an assessment path with a near-exclusive focus on toxicological hazard to the entity of value (shredding caddisfly, *Lepidostoma liba*). If, instead, they had focused on the pathway of exposure to define the specific stressor - Cry1A(b) protein in their study - and its environmental concentration, a conclusion as to no probable harm would have been more readily obtained.

### Uncertainty: variability and lack of knowledge

The analytical plan and subsequent risk findings need to consider uncertainty as an inherent part of the risk assessment. Both lack of knowledge and variability need to be specifically considered with respect to the risk characterization. Lack of knowledge can be reduced through data acquisition, but can never be eliminated as an unresolved residual in the determination of risk. Variability, on the other hand, is inherent to risk analysis which is comprised of measurements of parameters; it cannot be reduced but it can be better described with improved information [7]. For new technologies, such as transgenic biofeedstock crops released to the environment, the degree of uncertainty inherent in risk assessments will be relatively high but will be reduced over time as knowledge is accumulated. Improved knowledge over time is the reason why the iteration of risk assessment is necessary throughout the life cycle of any technology.

Addressing the uncertainty of the risk characterization should reflect the degree of understanding needed to describe behaviour of the transgenic crop within the bounds of a conservatively cast threshold for harm. Given that there is sufficient certainty that the behaviour of the transgenic crop does not exceed a conservative threshold that could lead to harm, greater precision is unnecessary for the purposes of risk assessment. For example, if we are sufficiently certain that a RQ is < 0.1, and this represents a threshold for concern, a more exact value of the RQ does not represent greater certainty in the risk finding for the purposes of decision-making.

Second order, or 'iatrogenic' risk, [7] can contribute uncertainty through either over analysing a negligible risk or failing to recognize a substantive risk. Problem definition attempts to eliminate missed risks (false negatives and surprises) by conservatively assuming, in the first instance, worst-case exposure scenarios [30]. Consequently, risk assessments are likely to include the characterization of a number of false-positive risks. The generation of false positives increases the resources and time needed to assess negligible risks and it can be managed through the use of a staged scheme of assessment that iterates through tiers. Since the outcome of any risk assessment is subject to reanalysis when new information is available, the iterative nature of the risk assessment allows for the problem to be reformulated for further analysis to reduce residual uncertainty from an earlier stage of assessment.

The concept of using a tiered system and recognized defaults for risk assessment is an important method for dealing with uncertainties [7]. Within the context of a tiered assessment paradigm, each iteration of problem formulation identifies the appropriate tier for analysis and, whenever possible, validated test systems and defaults for the analysis. Some aspects of uncertainty are addressed by using conservative assumptions within each tier of assessment. Also, since transgenic plants are assessed through a lines-of-evidence approach, it is important that the findings of any one line of analysis be consistent with respect to other evidence.

Use of comparative risk assessment is a further means whereby uncertainty is reduced by the consideration of carefully matched comparators (isogenic lines, cropping managements, environments) in order to focus on aspects of the comparison that represent substantive, consequential changes in the crop or system under consideration. By focusing on aspects of the crop or system that are changed for the transgenic biofeedstock crop relative to a baseline comparator, there is a reduction in the number of parameters and assumptions that must be considered. For instance, if the change brought about via plant transformation is to express a single unique protein in a plant, then the focus can be largely on the protein and its expression system, given that the principle of comparability (familiarity and substantial equivalence) has been addressed.

### The risk assessment-risk management interface

Increasing transparency in the risk assessment process by establishing problem context is integral to the relevance of risk assessments to both narrowly defined regulatory considerations and broader-based societal decision-making [42]. Transparency allows for risk management to be clearly distinguished from the risk assessment processes (Figure 1). Transgenic biofeedstock crop risk assessments can benefit from current best practice for science and decision-making at the risk management-risk assessment interface [7,30]. The problem context should include the recognition of iterative processes (including established tiers and defaults), the use of comparative approaches, and explicit consideration of uncertainty as part of the risk assessment framework. Since the acceptability of a given level of risk (including inherent uncertainty) is a matter of negotiation among different stakeholders, all of whom must recognize the impossibility of 'zero risk' for decision-making, the problem context should clearly establish the standard for decision-making such as 'reasonable certainty' or 'safe as' in a comparative sense. Finally, the problem context seeks to balance the need for transparency in the process with the recognition that certain data underpinning the assessment may be confidential.

Once the standard for decision-making is understood, the strength of risk assessment as originally detailed by the Red Book paradigm [11] can be more fully realized. We next consider various relevant transgenic biofeedstock case instances in order to better understand the opportunities and limitations for advancing risk assessments for novel agricultural biofeedstocks.

## Transgenic biofeedstock case instances

### *In-planta *bioprocessing enzymes

Early generation innovation for bioenergy crops has emphasized *in planta *production of key bioprocessing enzymes as a means to improve production efficiency and economy (as opposed to using microbial enzyme preparations) [4,43]. For instance, multiple crops have been bioengineered for producing glucanase [44-48], laccase [49] and amylase [50] for use in biofuels production. Plant-made transgenic enzymes may exhibit properties that confer increased environmental stability and this, along with the biophysicochemical attributes of many of these enzymes, make the nature and ramifications of their introduction to the environment and their potential occurrence in foods uncertain. This is especially so when the enzymes are intended for widespread environmental release. From an environmental perspective, there is no *a priori *reason to anticipate that environmentally stable enzymes will have direct effects to non-target organisms, but rather they may have indirect effects on critical ecological services such as nutrient cycling and decomposition. Thus, the need for environmental risk assessment is initiated from an ecological value context (Appendix 1). In terms of human health, confinement conditions and consequences of acute episodes of low-level occurrence in the food supply provide the relevant context for initiation of a food safety risk assessment, even though these products are intended for non-food uses [51].

The need for case-by-case approaches to risk assessment is shown for contrasting cases where bioprocessing enzymes are expressed in maize as aids for ethanol production. Highly thermostable forms of alpha-amylase and endoglucanase have been expressed in maize kernel starch endosperm [50], ubiquitously in roots and shoots [45] or in green tissues only [48] to affect improved economies and efficiencies of ethanol production. In each case, the potential for environmental exposure is an important aspect to understanding the risk to ecological services. The site and level of expression will determine the environmental loadings of these enzymes in plant residuals. Therefore, exposure characterization is useful for establishing the scope of concern regarding environmental release of a biofeedstock crop and, thus, can be used to guide problem formulation for refinement of the risk assessment. Approximately 1.3 × 10^5 ^hectares could be devoted to high amylase maize production in the USA, with environmental loadings of thermostable alpha-amylase expressed in grain conservatively estimated as 0.14 kg/ha for residues of high amylase maize production [52]. In contrast, when expression of recombinant thermostable endoglucanase is ubiquitous in both roots and shoots of transformed maize [45], there is the potential for its presence in maize stover harvested from millions of hectares in the USA. The unharvestable biomass from glucanase maize could lead to endoglucanase loadings in cropped fields of > 30 kg/ha; a loading estimate almost 50-fold greater than the comparable estimate for alpha-amylase from high amylase maize [52]. The differing magnitude of the environmental loading estimate is largely related to the differing sites of expression (grain versus biomass) in the plant which influences the potential residue left within the field. Thus, if the thermostable endoglucanase is expressed in green tissue only [48], the residuals introduced into the environment are substantially lowered. A further important consideration for the environmental exposure from plant-made industrial enzymes is the extent of exposure. For instance, with endoglucanase expressed in maize stover, production would be appreciably more than for the case where alpha-amylase is expressed only in grain and where confinement and channelling greatly restricts the area subject to annual production of this biofeedstock crop.

For plant recombinant enzymes, as represented by the forgoing cases, a tiered process for assessing environmental exposure and effect provides an efficient means to characterize risk in a manner proportional to the reasonably anticipated impact. For instance, a tiered process for discerning novel enzyme impacts to soil processes could proceed in a hierarchical fashion from (i) characterization of fundamental attributes of the molecule and its interaction with soil colloids; to (ii) characterization of chemical reactivity; (iii) determination of environmental degradation and persistence; and - as needed - culminate with (iv) impact assessment for critical soil processes. The overarching goal of such a tiered approach is to provide regulators and data developers with effective means whereby - through a stepwise process - the environmental fate and effects of diverse types of bioactive transgenic proteins introduced to the environment can be assessed with regard to impact on soil processes. Similar tiered processes also can be implemented for other aspects of the risk assessment. For instance, from the perspective of human health, an early tier focus on exposure characterization can contribute to the determination of the degree to which the consequence of admixture of the industrial product within the food supply should be evaluated in a higher tier assessment [25].

For these industrial enzyme cases, exposure characterization is a useful first step in problem scoping. However, since for these cases the problem formulation initiates from uncertainty regarding the impact to an ecological value (soil ecological services), exposure analysis alone will be insufficient to establish risk. Further problem definition can determine the appropriate analytical plan for risk characterization. The analytical plan for more fully characterizing risk in this instance would focus on measurements of the ecological value (the functional role of these enzymes in the soil ecosystem).

A limitation of many current studies ostensibly designed to address risks of transgenic plants to the environment is a tendency to focus on structural elements of the ecosystem, such as a specific non-target species, rather than on functional endpoints such as ecological services [53]. Thorough problem formulation as shown in this case helps to avoid this pitfall.

### Environmental stress tolerance

The use of transgenics to confer plant tolerance to environmental stresses, such as drought, chilling or salts, has been envisioned as a means to improve crop productivity [54] and numerous efforts to confer tolerance to environmental stress through gene expression, transcriptional regulation or signal transduction are underway [55-58]. Transgenic expression of regulatory proteins, enzymes, transporters and chaperones will be a key enabler for developing stress tolerant crops [57]. Improving plant stress tolerance is especially important with respect to biofeedstock crops, in view of the need for expanding crop production to marginal lands for both food and biofuel crop production; addressing environmental stress brought about through climate change; and assuring consistent, predicable biomass supplies for processing.

The risk assessment paradigm for stress tolerant transgenic crops is similar to that for existing transgenic crops. However, there is a recognized need for increased emphasis on aspects of the risk assessment that deal with invasiveness potential and the environmental and human health aspects of the alteration of plant composition [39]. Appropriate problem formulation provides the means whereby existing guidance for transgenic plant risk assessment can be shaped to address the specific context of the environmental release of a transgenic stress tolerant crop. This approach leads to a focused analytical plan to address specific knowledge gaps, rather than merely conducting a wide array of studies, as suggested by Strandberg *et al*. [39]. Nickson [34] developed an example of problem formulation consistent with environmental release of drought tolerant maize and concluded that the appropriate risk characterization would consider persistence, invasiveness and plant compositional profile. However, the simple definition of the high level phenotype in this instance as a 'drought tolerance trait' will probably prove too limiting for a robust risk assessment, since the nature of the change that confers drought tolerance may lead to differing risk perspectives. For instance, changing plant architecture to confer drought tolerance [59] would lead to a well-defined problem focusing risk assessment on the whole plant and the environment where it will occur and would logically emphasize questions of weediness or invasiveness. On the other hand, conferring drought tolerance through the alteration of stress-associated metabolism represents a major gap in understanding, requiring comprehensive profiling of stress-associated metabolites [55]. In this instance, problem formulation would entail identifying the appropriate comparative approach and analysis plan for the risk assessment in order to consider more comprehensively the consequences of the altered plant metabolite profile with respect to food safety and non-target risks, in addition to weediness or invasiveness issues.

*Eucalyptus *genetically engineered for freeze tolerance and targeted for introduction as a biomass crop in forest plantations in the Southeastern USA exemplifies a transgenic biofeedstock crop where the risk assessment focuses on the invasiveness of the whole plant [60]. The preliminary assessment has concluded the non-transformed hybrid (*Eucaliptus grandis *× *E. urophylla*) has been cultivated in southern Florida where it is a familiar, non-invasive species. This finding, however, is not consistent with the assignment of *E. grandis *to an invasiveness watch list for Florida [61]. As a precursor to a full environmental risk assessment, the comparability of the freeze-tolerant transgenic hybrid to the non-transgenic hybrid must be evaluated in order to establish that, other than for the tolerance to freezing conveyed through expression of the *Arabidopsis *C-Repeat Binding Factor [62], the transgenic plant is substantially the same as the familiar non-transgenic hybrid. Studies to establish comparability in terms of invasive characteristics will prove challenging, as the non-transgenic hybrid will not persist in the more northerly environments where the transgenic hybrid is intended for release. As a strategy for the management of risk associated with the field testing of the transgenic hybrid, further genetic modification for control of pollen flow is envisioned [62]. In addition to regulatory-driven consideration of invasiveness, the potential for *Eucalyptus *plantations to supplant existing tree plantations may raise questions regarding the ecological community structure [63]. These issues of changes in large-scale land use lie outside the development process but may need to be addressed within a broader decision-making context.

### Plant multi-products

Numerous life-cycle analyses have considered the various costs of biofuel (ethanol and biodiesel) production [64,65]. And many have addressed the impact of feed by-products of the biofuel industry, such as dried distillers grains with solubles (known as DDGS) and biodiesel by-products (known as BDBP; for example soy or rapeseed meal) [66,67]. Less consideration has been given to the economics of value-added co-products such as plastic composites, therapeutics, or industrial chemicals [68]. Yet the sustainability of the bioeconomy will depend, in part, on moving the focus of plants as biomass to plants as biorefineries for versatile multi-product production [43].

Co-production and extraction of heterologous proteins from biofeedstock crops is a critical consideration for the sustainability of cost-effective biofuels production [69]. Heterologous protein production in plants to provide industrial and therapeutic compounds is a well-proven concept with considerable commercial promise, provided that concerns regarding environmental containment and the channelling of downstream processing are effectively addressed [70,71]. Although it makes sense to use high biomass producing crops already devoted to biofuels production as platforms for multi-product production [69], it may be more practically feasible to focus on developing plants that will produce industrial and therapeutic compounds with a secondary use of the biomass for biofuel production [4].

Human health and environmental risk assessment are critical to the development of plant multi-product concepts, in view of the consequences arising from their in-field confinement and downstream processing, segregation and channelling [72]. Again, the risk assessment frameworks for plant biorefineries bear similarities with those for conventional transgenic plants, but emphasis shifts to the aggregate risk arising from individual products of expression and their potential interactions. For instance, current generation crops genetically engineered for herbicide and pest resistance include trait stacks that express combinations of proteins which must be evaluated in terms of the aggregate risk of the co-occurring traits [73]. The principal risk consideration for multiple traits, compared to an individual trait, is the possibility for negative synergistic effects and their consequences (as, for instance, in a broader spectrum of non-target effects than would be evident from any one trait alone). The data needs for the risk assessment of stacked traits that result from conventional crosses between transgenic lines representing unique transformation events (breeding stacks) is basically the same as for multiple traits arising from a single transformation event (transformation stacks). However, the way in which data are generated will differ. Risk assessments for breeding stacks evaluate the traits separately and then consider the aggregate consequences of their combination. In risk assessments for the transformation stack the aggregated effect is directly evaluated. For conventional transgenic crops, unique multi-trait risk assessment considerations relate, for the most part, to environmental effects. For multi-trait biofeedstock crops there will be an added emphasis on exposure characterization in order to understand the way that various bioproducts are appropriately processed and segregated. Risk assessment will seek to understand the consequences of unintended presence if, for example, a product intended for industrial or therapeutic use were to occur in foods [25].

### Plant domestication

The long-term success in developing sustainable bioenergy resources is frequently tied to perennial herbaceous and woody plants such as switchgrass (*Panicum virgatum*) and poplar (*Populus*) for ethanol production [4,74,75] or jatropha (*Jatropha curcas*) for biodiesel production [76]. The targeted attributes for an ideal bioenergy crop vary, depending on whether the objective is a dedicated biofeedstock crop or a food and fuel crop such as maize or soybean (*Glycine max*) [3]. In those cases where range and forest plants are targeted as dedicated biofeedstock crops there will be a need for domestication in order to improve agronomic performance, uniformity, quality and productivity. These crops will also need to undergo compositional modifications, for instance to better affect the conversion of lignin, cellulose and other cell wall polysaccharides to ethanol or to improve yield and quality of oils [4,77]. Genetic engineering, in conjunction with genomics and conventional breeding, will allow for the accelerated domestication of plants for specific use as biofeedstock crops [4,75].

Issues regarding the weediness and invasiveness of the transgenic plant, as well as of gene flow into wild populations, will be of paramount concern in risk assessments of forest and range plants which are domesticated and otherwise modified for use as biofeedstock crops. Given the reality of gene flow as a natural phenomenon, emphasis in the risk assessment should be placed on the consequences of transgene occurrence within native populations. The consequences will vary, dependent on the receiving environment and the ecological value of receptor populations. For considerations of weediness, invasiveness and gene flow there are effective models for analysis of plant introductions [78], invasive plants [79] and risk assessments for current generation transgenic crops [80] which can be readily applied to transgenic biofeedstock crops. As shown in current generation examples of transgenic crops, special emphasis must be given in formulating risk assessments which are specific to the region of environmental release. For instance, the environmental release of transgenic maize events throughout the Midwestern USA entails little consequence regarding gene flow into the environment, since there are no wild or related species present [80]. When, however, transgenic maize release is considered for Mexico, there is heightened concern as to the consequences of gene flow as both wild relatives and domestic land races comprise a unique reservoir of genetic resources within the crop's centre of origin [81].

Issues of gene flow are not restricted to the transgenic biofeedstock crop. For instance, for switchgrass introduced into the upper midwestern USA many of the same questions of gene flow impact to local ecotypes exist for a non-transgenic southern variety of switchgrass as there will for a transgenic switchgrass variety. Regulatory statutes differ, however, such that, while the consequences of gene flow from the transgenic variety will always be explicitly considered in a regulatory-compliant environmental risk assessments, the need to do a similar assessment for a newly introduced non-transgenic biofeedstock crop will vary depending on the regulatory jurisdiction. Furthermore, consequences of crop-to-crop gene flow may be relevant in certain risk assessments and not others, depending on the operative statute. Nevertheless, the fore-mentioned analytical models are able to address gene flow and its consequences in any of these situations.

## Conclusion

The regulatory safety and public acceptance of a given transgenic biofeedstock crop are informed through the science-based process of risk assessment. Frameworks for the risk assessment of transgenic plants are becoming well-established and provide a logical approach for considering the human health and environmental consequences of various sustainable biofeedstock crops. In applying these frameworks to biofeedstock crops, special emphasis is needed regarding comparative risks of new versus established agronomic systems and for environmental release within a given region. In addition, aggregate risks arising from use of crops as multi-product production platforms will need to be more comprehensively considered and developed. The strength of risk assessments for transgenic biofeedstock crops depends on flexibility for case-specific determinations which are designed on the basis of problem formulation and use sequential approaches for data development and risk characterization.

## Abbreviations

EC: effect concentration; EEC: estimated environmental concentration; NOAEC: no observed adverse effects; RQ: risk quotient.

## Competing interests

The author declares that they have no competing interests.

## Appendix

### Appendix 1. Problem statement and risk hypothesis examples for transgenic crops as initiated from various perspectives of potential harm

### Stressor-initiated [23,32]

#### Problem statement

Proteins from the Cry1 class of delta-endotoxins of *Bacillus thuringiensis *are selectively active on Lepidoptera. Cry1A(b) protein is expressed in maize for the targeted control of European maize borer (*Ostrinia nubilalis *(Hubner)) and may harm sensitive non-target Lepidoptera if exposed in and around fields of Cry1A(b) maize. Monarch butterfly (*Danaus plexippus*) occurs in and around maize fields where it may be exposed to Cry1A(b) maize pollen in its diet.

#### Risk hypothesis

Dietary exposure to Cry1A(b) maize pollen will not adversely affect Monarch populations in and around maize fields to a greater extent than does non-transgenic maize pollen when exposure occurs to at environmentally relevant concentrations.

#### Testing hypothesis and rationale

Hazard is established on the basis of stressor class. Effect characterization - Tier 1: EC_50 _for purified toxins incorporated into an artificial diet relative to control; Tier 2: Dose-response for larval weight for Cry1A(b) pollen present on food source (milkweed) relative to a non-transgenic control. Exposure characterization - Estimated environmental concentration (EEC) for Cry1A(b) present in pollen contaminating milkweed leaves. Risk characterization - Risk is formulated as a risk quotient (RQ = EEC/EC_50_) with a regulatory level of concern being RQ > 0.1.

### Effect-initiated [31,82]

#### Problem statement

Where transgenic insect-resistant (IR) crops are grown, field monitoring shows shifts in beneficial insect abundance relative to non-transgenic fields. Agroecosystems where transgenic IR crops are grown may have trophic level effects affecting occurrence and distribution of beneficial insects relative to comparable agroecosystems where transgenic IR crops do not occur. Ladybird beetle (*Coleomegilla maculata *(De Geer)) may be adversely affected when feeding on prey that have ingested toxins expressed in potatoes expressing Cry3A toxin. Development and fecundity may additionally be related to feeding on pollen as a supplemental food source.

#### Risk hypothesis

Cry3A-intoxicated Colorado potato beetle (*Leptinotarsa decemlineata *(Say)) and Cry3A potato pollen can be eaten by ladybird beetle without adverse effects on their survival or predation potential relative to eating non-intoxicated Colorado potato beetle and non-transgenic potato pollen.

#### Testing hypothesis and rationale

The prey ingests toxin through feeding on Cry3A potatoes and is susceptible to the toxin. Predator ingestion of, and susceptibility to, Cry3A may come through direct pollen exposure and/or prey-mediated effects.

### Exposure-initiated [83,84]

#### Problem statement

Crop improvement through RNA interference (RNAi) may introduce novel RNA sequences into the environments where RNAi crops occur. Persistence of these RNA sequences may create off-target effects in receiving environments.

#### Risk hypothesis

Persistence of plant-expressed RNAi in the soil environment is no different than for wild-type short length RNA sequences that are introduced to the soil environment.

#### Testing hypothesis and rationale

There is uncertainty as to the nature of off-target unintended effects that could occur. Determination as to whether there is a causal pathway for exposure to soil biota addresses the extent to which unintended effects are possible. Lack of persistence will significantly reduce probable harm to off-target biota.

### Ecological value-initiated [19,48]

#### Problem statement

Extracellular soil enzymes provide important ecological services relative to the dynamics of organic matter mineralization and carbon substrate stabilization. Soil cellulase enzymes, in particular, are important for degradation of plant cellulose residues and therefore impact organic matter mineralization rates which, in turn, influence soil carbon stabilization. Thermostable endo-1,4-β-glucanase expressed in maize may increase activity in the degradation of plant residues incorporated to soil to affect the dynamics of soil carbon stabilization.

#### Risk hypothesis

Rates of organic matter mineralization in soil receiving plant residues from high glucanase maize will not differ from that observed for wild type enzymes when considered under environmentally relevant conditions and concentrations.

#### Testing hypothesis and rationale

Uncertainty as to consequence, so seek a determination of no difference between the transgenic and wild-type enzyme, in order to be conservatively protective of ecological-value.
